# Levels and clinical significance of the m6A methyltransferase METTL14 in patients with coronary heart disease

**DOI:** 10.3389/fcvm.2023.1167132

**Published:** 2023-06-27

**Authors:** Fengxia Guo, Mei He, Bing Hu, Gang Li

**Affiliations:** ^1^ Department of Clinical Laboratory, Henan Provincial People’s Hospital; People’s Hospital of Zhengzhou University, Zhengzhou, China; ^2^ Zhengzhou Key Laboratory, Zhengzhou No. 7 People’s Hospital, Zhengzhou, China; ^3^ Department of Clinical Laboratory, Affiliated Cancer Hospital of Zhengzhou University, Zhengzhou, China

**Keywords:** coronary heart disease, m6A methylation, METTL14, inflammatory markers, atherosclerosis

## Abstract

**Objective:**

To investigate the association of methyltransferase-like protein 14 (METTL14) expression with coronary heart disease (CHD).

**Methods:**

Three hundred and sixteen patients who attended Henan Provincial People's Hospital between June 2019 and February 2021 with principal symptoms of pain or tightness in the chest and who underwent coronary angiography for definitive diagnosis were enrolled. The uric acid, TG, TC, LDL-C, HDL-C, apolipoprotein A1, free fatty acid, lipoprotein a, homocysteine, CRP, and SAA levels were examined. The levels of METTL14, TNF-α, MCP-1, VCAM-1, ICAM-1, and IL-6 were evaluated by ELISA.

**Results:**

Patients with CHD had significantly higher m6A methyltransferase activity. In addition, the incidence of diabetes and hypertension, as well as the concentrations of TC, CRP, and SAA were higher in CHD patients. Patients with coronary lesion branches also had significantly increased TG, LDL-C, CRP, and SAA levels. TNF-α, MCP-1, VCAM-1, ICAM-1, and IL-6 expression was also markedly increased in the CHD group (*P* < 0.001) as was the expression of METTL14 (*P* < 0.001). The METTL14 expression levels also differed significantly in relation to the number of branches with lesions (*P* < 0.01) and were correlated with SAA, VCAM-1, ICAM-1, IL-6, and the Gensini score. ROC curve analyses of METTL14 in CHD indicated an AUC of 0.881 (0.679, 0.894) with a cut-off value of 342.37, a sensitivity of 77%, and a specificity of 84%. MCP-1, VCAM-1, IL-6, SAA, and METTL14 were found to independently predict CHD risk.

**Conclusions:**

METTL14 levels were found to be positively associated with inflammatory markers and to be an independent predictor of CHD risk.

## 1. Introduction

Coronary heart disease (CHD) is increasing in incidence. The current tests used to assist in the diagnosis of CHD have limited specificity and sensitivity and are invasive; thus, the identification of specific biomarkers is important for the early detection of CHD. CHD is both chronic and progressive and evidence suggests a close association between CHD and inflammation, which can be assessed by a variety of inflammatory indicators, suggesting that the levels of these indicators may be useful for preventing, diagnosing, and treating CHD (1–4). Thus, a comprehensive analysis of potential indicators and biomarkers for CHD development and progression would be highly useful for the assessment of CHD risk.

The importance of epigenetic modification is increasingly recognized. Nucleic acid methylation is a major form of epigenetic modification, with N6-methyladenosine (m6A) methylation representing approximately 80% of RNA modifications in eukaryotes (5). m6A methylation is controlled by a series of enzymes, specifically, the “writers” or m6A methyltransferases, such as METTL3/14, VIRMA, RBM15/15B, WTAP, and ZC3H13, responsible for the methylation, the “erasers” or demethylases, such as ALKBH5, FTO, and ALKBH3, that remove the modification, and “readers”, such as hnRNP, YTHDF1/2/3, eIF3, and IGF2BP1/2/3 (6, 7). Evidence suggests the close involvement of m6A methylation in regulating RNA function and these modifications have been associated with pathological processes in in physiological and pathological processes of cardiovascular diseases (8). Methyltransferase-like 14 (METTL14), a well-known m6A writer protein, widely participated in the progression of major diseases, such as cardiovascular pathogenesis (9). In addition, METTL14 plays an important role in maintaining cardiac homeostasis (10). Knocking down METTL14 could inhibit the development of atherosclerosis in high-fat diet-treated APOE-/- mice (11). Here, the clinical significance of alterations in the METTL14 levels of CHD patients was investigated to clarify the association between METTL14 and the severity of CHD. It is hoped that these findings will suggest new directions for CHD prevention and treatment.

## 2. Study participants and methods

### 2.1. Patients

A total of 316 patients who attended Henan Provincial People's Hospital between June 2019 and February 2021 with principal symptoms of pain or tightness in the chest and who received coronary angiography for definitive diagnosis were recruited. The patients were allocated to a CHD group and a control group based on the angiographic findings. Patients in the control group had no stenosis of the coronary arteries or only myocardial bridge alterations (i.e., congenital abnormal coronary artery development where a part of the coronary artery crosses the myocardium). The inclusion criterion for the CHD group was the presence of a >50% stenosis in at least one coronary artery (left main, left circumflex, left anterior descending, or right coronary artery). Patients who had undergone earlier coronary angiography and coronary artery bypass grafting were excluded, as were those with hematological disorders, tumors, severe liver and renal insufficiencies, acute or chronic infectious diseases, active bleeding from all causes, peripheral vascular disease, diabetes mellitus, cardiac arrhythmia, or chronic obstructive pulmonary disease. The study was approved by the Ethics Committee of the hospital, and all participants provided written informed consent.

### 2.2. Collection and analysis of blood samples

Five-milliliter venous blood samples were collected after an overnight fast. After centrifugation (3,000 rpm, 15 min), the uric acid, TG, TC, LDL-C, free fatty acid, lipoprotein a, homocysteine, HDL-C, Apolipoprotein A1 (ApoA1), CRP, and plasma amyloid A (SAA) levels were determined.

### 2.3. Methods

#### 2.3.1 ELISA

ELISA kits (Abcam, Cambridge, UK) were used to measure the plasma levels of METTL14, TNF-α, MCP-1, VCAM-, ICAM-1, and IL-6.

#### 2.3.2. Methylase activity assay

The m6A methylase activity was determined using an Epigenase m6A Methylase Activity Assay kit (Epigentek, NY, USA).

#### 2.3.3. qRT-PCT

Total RNA was isolated from plasma using a TRIzol kit (Invitrogen, Carlsbad, CA, USA). The RNA concentration was measured with a spectrophotometer (NanoDrop® 2000; Thermo Fisher Scientific, Waltham, MA, USA). All experimental procedures were performed according to the manufacturer's instructions. Ploidy differences in expression levels were determined using the 2-ΔΔCt method.

METTL14 Forward: 5′-GTT GGA ACA TGG ATA GCC GC-3′; Reverse: 5′-CAA TGC TGT CGG CAC TTT CA-3′.

GAPDH Forward:5′-GGTGGTCTCCTCTGACTTCAA-3′; Reverse: 5′-GTTGCTGTAGCCAAATTCGTTGT-3′.

FTO forward: 5′-CTTCACCAAGGAGACTGCTATTTC; Reverse: 5′-CAAGGTTCCTGTTGAGCACTCTG-3′.

#### 2.3.4. Gensini score

The Gensini score equals the sum of all segment scores (each segment score equals a segment weighting factor multiplied by a severity score). The segment weighting factors range from 0.5 to 5.0. The severity scores reflecting the specific percentage of luminal diameter reduction in the coronary artery segment are 32 for 100%, 16 for 99%, 8 for 90%, 4 for 75%, 2 for 50%, and 1 for 25% reduction. Thus, segments supplying a larger area of the myocardium are more heavily weighted and the highest scores are associated with multiple severe proximal lesions. Scoring was performed according to internationally recognized methods and the score of the individual patients represented the summed scores for each branch (12).

**Table:** 

Narrowness	Score	Lesion	Score
1%–25%	1	Left main stem	5
26%–50%	2	Left anterior descending branch or left gyral branch	2.5
51%–75%	4	Middle left anterior descending branch	1.5
76%–90%	8	Distal segment of the left anterior descending branch	1.0
91%–99%	16	Middle and distal left gyral branch	1.0
Fully closed	32	Right coronary artery	1
		Small branch	0.5

### 2.4. Statistical analysis

Data were analyzed using SPSS 23.0. Data distributions were assessed by Kolmogorov–Smirnov tests and normally distributed data were expressed as means ± standard deviations and compared using independent-sample *t*-tests for two groups and one-way ANOVAs for multiple groups. Data that did not conform to a normal distribution were expressed as medians (interquartile spacing) and compared with the Mann–Whitney *U* rank-sum test for two groups and the Kruskal–Wallis rank-sum test for multiple groups. Associations between variables were assessed by Pearson or Spearman correlation analysis, and the sensitivity and specificity of METTL14 were evaluated by ROC curves and logistic regression analysis of risk factors for CHD. The discrimination ability of the logistic regression model was assessed by estimating the area under the receiver operating characteristic (ROC) curve. Model calibration was assessed using the Hosmer–Lemeshow test for good-ness of fit. Differences were statistically significant at *P* < 0.05.

## 3. Results

### 3.1. Activity of the methyltransferase are significantly raised in CHD

**Figure 1:**
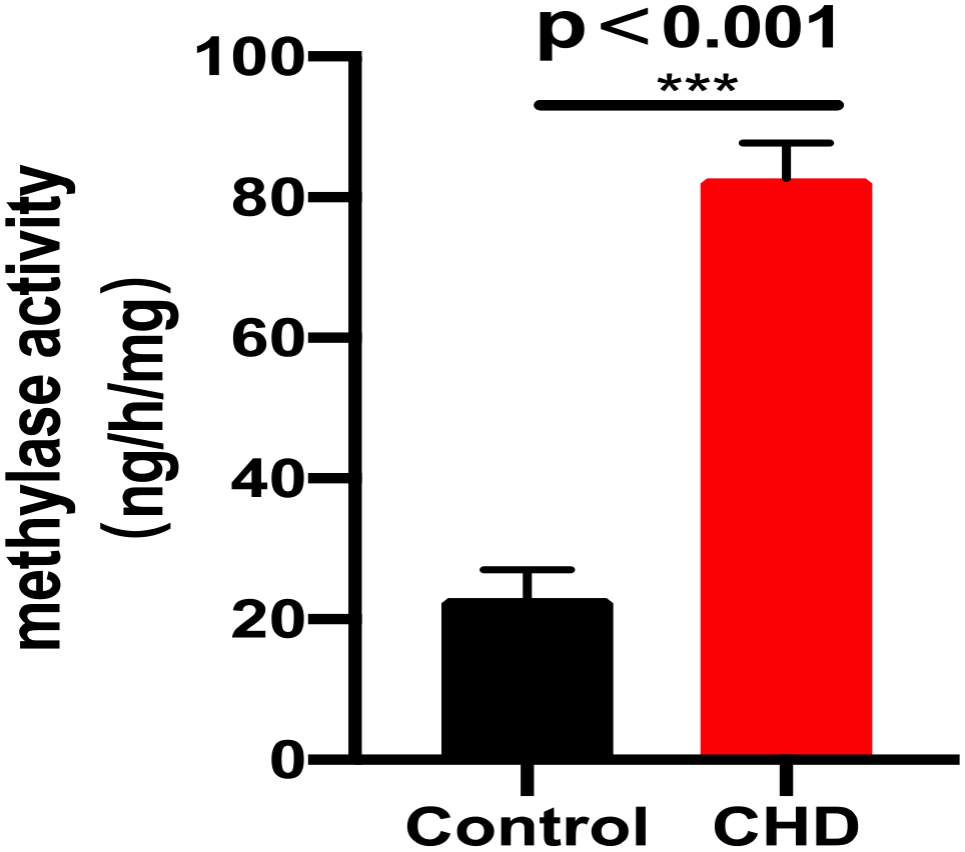
The activity of methyltransferase.

**Figure 2:**
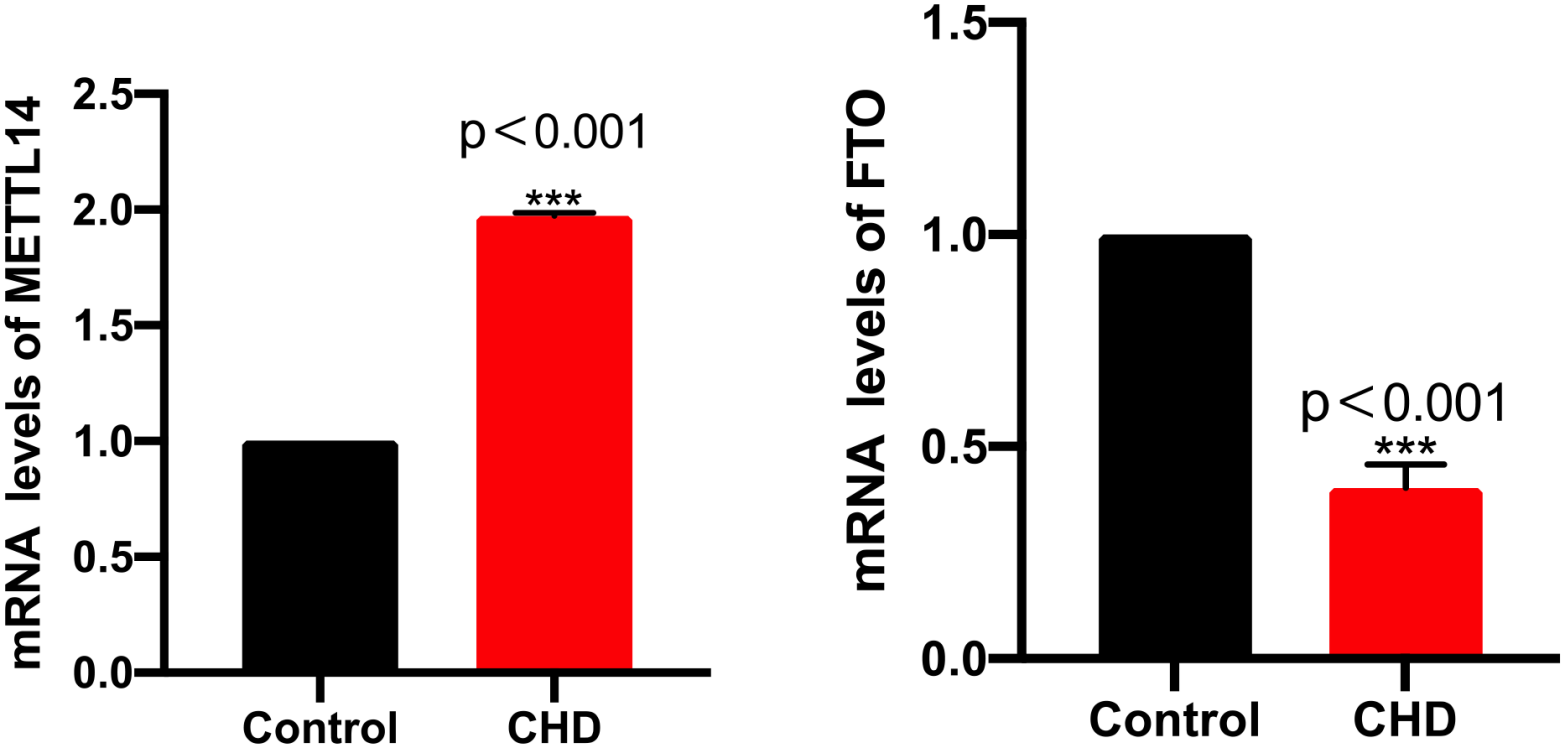
The mRNA levels of METTL14 and FTO.

Firstly, we used ELISA to determine the activity of methyltransferase in the plasma of patients with CHD, finding the activity of the methyltransferase significantly raised in CHD patients relative to the controls (Figure 1). In addition, the mRNA levels of METTL14 were markedly increased while the FTO levels were significantly reduced in the CHD group (Figure 2).

### 3.2. Comparison of patient characteristics between the groups

No significant differences were observed between the groups in relation to age, sex, smoking, drinking, UA, TC, TG, LDL-C, FFA, LPa, HCY, HDL-C, or ApoA1 (*P* > 0.05). However, as shown in Table 1, the incidence of diabetes and hypertension, as well as the concentrations of TC, CRP, and SAA were higher in CHD patients.

**Table 1:** Characteristics of patients in the two groups.

	Control (*n* = 100)	CHD (*n* = 216)	*P*
Age (year)	55.38 ± 10.02	56.03 ± 5.19	0.359
Sex (Male/Female)	48/52	97/119	0.054
Hypertension [case (%)]	37 (37%)	138 (63.8%)	0.001
Diabetes [case (%)]	42 (42%)	103 (47.6)	0.034
Smoking [case (%)]	37 (37%)	78 (36.1%)	0.421
Drinking [case (%)]	34 (34%)	69 (31.9%)	0.390
UA (umol/L)	302.48 ± 79.55	322 ± 67.5	0.311
TG (mmol/L)	1.34 (0.88,2.14)	1.39 (1.21,1.95)	0.688
TC (mmol/L)	1.03 ± 0.79	4.1 ± 0.83	0.001
LDL-C (mmol/L)	2.88 ± 0.78	2.96 ± 0.84	0.603
FFA (mmol/L)	0.51 ± 0.34	0.68 ± 0.49	0.062
Lpa (mg/dl)	129.4 (72,321.3)	141.9 (69.46,423.4)	0.087
HCY (umol/L)	10.3 (9.3,13)	10.7 (9.6,15.9)	0.059
HDL-C (mmol/L)	1.13 ± 0.47	1.05 ± 0.22	0.074
ApoA1 (g/L)	1.43 ± 0.38	1.45 ± 0.30	0.183
CRP (mg/dl)	5.90 ± 3.18	30.28 ± 6.7	<0.001
SAA	4.35 ± 0.25	23 ± 2.21	<0.001

### 3.3. Numbers of coronary branches with lesions

Patients with CHD were then classified by the number of coronary branches containing lesions. Seventy-two patients had a single lesion in one branch, 80 had lesions in two branches, and 64 had lesions in three branches. Analysis of the clinical parameters of the patients in these different subgroups showed no inter-group differences in terms of age, sex, hypertension, diabetes, smoking, alcohol consumption, LDL-C, FFA, LPa, HCY, HDL-C, and ApoA1 while significant differences were observed for TG, LDL-C, CRP, and SAA (Table 2).

**Table 2:** Association between the number of coronary branches with lesions and clinical parameters in CHD patients.

	1 sticks (*n* = 72)	2 sticks (*n* = 80)	3 sticks (*n* = 64)	*P*
Age (year)	55.48 ± 5.93	55.8 ± 6.29	56.01 ± 4.38	0.403
Sex (Male/Female)	37/35	38/42	34/30	0.078
Hypertension [case (%)]	25 (34.7%)	30 (37.5%)	21 (32.8%)	0.055
Diabetes [case (%)]	21 (29.1%)	25 (31.2%)	22 (34.3%)	0.451
Smoking [case (%)]	24 (33.3%)	30 (37.5%)	24 (37.5%)	0.053
Drinking [case (%)]	20 (27.7%)	27 (33.7%)	21 (32.8%)	0.064
TG (mmol/L)	1.17 (1.12,1.5)	1.19 (0.89,1.6)	2.57 (1.32,2.99)	0.028
TC (mmol/L)	4.34 ± 0.58	4.46 ± 0.04	4.82 ± 0.75	0.348
LDL-C (mmol/L)	2.17 ± 0.71	3.02 ± 1.19	3.35 ± 0.23	0.017
FFA (mmol/L)	0.65 ± 0.31	0.69 ± 0.28	0.62 ± 0.34	0.178
LPa (mg/dl)	109.4 (73,205.4)	120.5 (84.3,450.9)	113.9 (69.6,423.4)	0.058
HCY (umol/L)	11.2 (8.7,18.4)	11.9 (9,21)	10 (8.7,17.6)	0.231
HDL-C (mmol/L)	1.01 ± 0.28	1.04 ± 0.39	1.12 ± 0.46	0.129
ApoA1 (g/L)	1.39 ± 0.42	1.45 ± 0.64	1.43 ± 0.29	0.274
CRP (mg/dl)	13.3 ± 4.39	29.5 ± 3.43	36.5 ± 5.4	0.001
SAA	19.3 ± 2.12	45.2 ± 1.38	88.7 ± 0.12	0.001

### 3.4. Levels of inflammatory factors in patients with CHD

It was observed that the contents of TNF-α, VCAM-1, ICAM-1, MCP-1, and IL-6, measured by ELISA, were significantly raised in the CHD group in comparison with the controls (*P* < 0.001) (Table 3).

**Table 3:** Levels of inflammatory factors.

	Control (*n* = 100) P50 (P25, P75)	CHD (*n* = 216) P50 (P25, P75)	*P*
TNF-α (pg/ml)	197 (95.23, 294.39)	403.5 (209.2, 488.90)	<0.001
MCP-1 (pg/ml)	302 (139.20, 388.47)	537 (219.3, 573.9)	<0.001
VCAM-1 (pg/ml)	153.6 (142.29, 232.3)	340.8 (267.4, 473.98)	<0.001
ICAM-1 (pg/ml)	109 (78.38, 236.4)	302.3 (148.3, 335.39)	<0.001
IL-6 (pg/ml)	174.3 (133.2, 289.3)	392.4 (212.3, 478.2)	<0.001

### 3.5. METTL14 levels in the CHD group

METTL14 levels were observed to be markedly increased in the CHD group relative to the controls (*P* < 0.001) (Table 4).

**Table 4:** METTL14 in the two groups.

	Control	CHD	*P*
METTL14 (pg/ml)	123.39 (112.67, 298.45)	438.17 (239.04, 468.23)	<0.001

### 3.6. METTL14 levels in relation to lesioned branches

Patients with CHD were grouped as described above based on the number of branches containing coronary lesions. The groups were defined as the 1-branch lesion (*n* = 72), 2-branch lesion (*n* = 80), and 3-branch lesion (*n* = 64) groups. The control was the 0-branch lesion group. The results are shown in Table 5.

**Table 5:** Levels of METTL14 in relation to the number of branches containing lesions.

Number of sticks	METTL14
0	123.4 (98.3,147.9)^a,b,c^
1	281 (234.5, 318.2)^a,b,d^
2	367.4 (325.8, 390.2)^a,c,d^
3	490.2 (436.18, 576.32)^b,c,d^

^a^ Compared with the 0-branch lesion group, *P* < 0.01 was statistically significant.

^b^ Compared with the 1-branch lesion group, *P* < 0.01 was statistically significant.

^c^ Compared with the 2-branch lesion group, *P* < 0.01 was statistically significant.

^d^ Compared with the 3-branch lesion group, *P* < 0.01 was statistically significant.

### 3.7. Associations between METTL14 levels and clinical characteristics of CHD patients

There was no correlation between METTL14 and age, sex, diabetes, hypertension, alcohol consumption, smoking, TG, TC, LDL-C, FFAs, LPa, HCY, HDL-C, or ApoA1 (*P* > 0.05). A significant association, however, was observed with SAA (*P* < 0.01) (Table 6).

**Table 6:** Relationships between METTL14 and clinical characteristics.

	METTL14	*P*
	R	
Age	0.045	0.509
Sex	−0.74	0.083
Hypertension	0.015	0.783
Diabetes	0.098	0.054
Smoking	−0.018	0.801
Drinking	0.023	0.612
TG	0.271	0.064
TC	0.382	0.078
LDL-C	0.641	0.392
FFA	−0.029	0.075
LPa	0.218	0.175
HCY	0.193	0.204
HDL-C	0.143	0.169
ApoA1	−0.292	0.403
SAA	0.012	0.03

R denotes correlation coefficient.

### 3.8. Associations between METTL14 and inflammatory factor levels and Gensini scores in CHD patients

METTL14 levels were significantly linked to those of VCAM-1, ICAM-1, SAA, and IL-6, as well as the Gensini scores in the CHD group (Table 7).

**Table 7:** Correlations of METTL14 with TNF-α, MCP-1, VCAM-1, ICAM-1, IL-6 and gensini scores.

	METTL14	*P*
	R	
TNF-α	0.372	0.062
MCP-1	0.4830	0.0594
VCAM-1	0.075	0.013
ICAM-1	0.05	0.029
IL-6	0.39	0.003
SAA	0.013	0.011
Gensini	0.493	0.027

### 3.9. ROC curve analysis of METTL14 sensitivity and specificity

The cut-off values and sensitivity and specificity according to the maximum Jorden index were determined from the ROC curves. The results showed that METTL14 had a cut-off value of 342.37, a sensitivity of 77%, a specificity of 84%, and an AUC of 0.881 (0.679, 0.894).

### 3.10. Binary logistic regression

In the regression analysis, CHD was set as the dependent variable with inflammatory factor and METTL14 levels as independent variables. The analysis showed that MCP-1, VCAM-1, IL-6, SAA, and METTL14 were independent risk factors for CHD (*P* < 0.05) (Table 8).

**Table 8:** Logistic regression analysis of METTL14 in CHD.

	β	Sx	Wald	RR (95% CI)	*P*
TNF-α	0.07	0.01	10.738	1.0007 (1.004,1.015)	0.312
MCP-1	0.05	0.03	10.487	1.005 (1.003,1.040)	<0.001
VCAM-1	0.09	0.04	11.783	1.013 (1.005,1.025)	<0.001
ICAM-1	0.05	0.02	1.291	1.003 (0.999,1.015)	0.382
IL-6	0.08	0.02	10.649	1.008 (1.005,1.023)	<0.001
SAA	0.04	0.04	3.204	1.019 (1.004,1.027)	<0.001
METTL14	0.05	0.03	13.08	1.002 (1.001,1.029)	<0.001

## 4. Discussion

Although the specific risk factors for CHD have not been fully elucidated, it is known that factors such as hypertension, hyperlipidemia, diabetes, and inflammation are associated with its development (13–15). A characteristic feature of CHD is the presence of atherosclerotic plaques, formed by a combination of lipid, calcium, and inflammation-associated cells (16, 17). The presence of plaque narrows the lumen of the artery, and, in the case of coronary arteries, can lead to the development of angina, either persistent or episodic. Plaque rupture can lead to the development of blood clots which can cause myocardial infarction through blockage of the vessels. Atherosclerosis is also linked with inflammation. It is thus possible to assess the severity and progression of CHD by measuring the levels of specific inflammatory indicators (18). Here, it was found that the presence of hypertension and diabetes, as well as the levels of TC, CRP, and SAA, were significantly associated with CHD. This suggests that both hyperlipidemia and the inflammatory response are closely associated with CHD pathogenesis, as has been found in earlier studies (17, 18).

Recent evidence has indicated that epigenetic modifications are associated with both the initiation and subsequent promotion of atherosclerosis, playing important parts in the development of atherosclerotic plaque. This suggests the potential significance of using markers of epigenetic modifications as indicators or biomarkers for CHD risk and progression (19). This appears to be the first investigation of the role of the methyltransferase METTL14 in CHD, and demonstrated that METTL14 levels were markedly raised in the sera of patients with CHD. However, the METTL14 levels were not found to be linked to either the TC or TG levels, which were used in the inclusion criteria.

Studies have shown that the m6A methylation process affects various types of cells, including those associated with blood vessels, such as vascular endothelial and smooth muscle cells, as well as macrophages, and that changes in methylation levels contribute to the pathogenesis of atherosclerosis. METTL14 is documented to methylate pri-miR-19a and promote the processing of the mature miR-19a, stimulating the proliferation and invasion of atherosclerotic vascular endothelial cells, indicating that the METTL14/m6A/miR-19a axis may represent a novel target for anti-atherosclerosis treatment (20). In addition, METTL14 reduction inhibits the endothelial cell inflammatory response, thereby preventing atherosclerotic plaque formation (21). A study using mass spectrometry to analyze m6A levels in non-atherosclerotic arterial and carotid atherosclerotic tissues found that m6A methylesterase and demethylase levels were altered in atherosclerotic tissues (22). More importantly, knockdown of METTL14 inhibited the m6A modification of FOXO1 and decreased FOXO1 expression to suppress the endothelial inflammatory response and atherosclerotic plaque formation (21). It has also been shown that METTL14 promotes inflammatory responses in atherosclerosis-associated macrophages via NF-κB/IL-6 signaling (23). WTAP promotes myocardial I/R injury through promoting ER stress and cell apoptosis by regulating m6A modification of ATF4 mRNA (24). NCBP3, a novel hypoxia-specific response protein, functions as a scaffold for the coordination of METTL3 and eIF4A2 for enhancing gene translation by m6A RNA methylation in cardiomyocytes subjected to hypoxic stress (25). Moreover, METTL14 promotes the renal ischemia-reperfusion injury (IRI) via suppressing YAP1 pathway (26). UCHL5 modified by METTL14/YTHDF1 axis could facilitate the inflammation and vascular remodeling in atherosclerosis by activating the NLRP3 inflammasome (27).

Here, plasma concentrations of inflammatory factors were analyzed by ELISA, showing that CHD patients had significantly elevated concentrations of TNF-α, MCP-1, ICAM-1, VCAM-1, and IL-6, which is consistent with previous findings (28–31). Notably, METTL14 levels were found to be significantly associated with those of VCAM-1, IL-6, ICAM-1, and SAA, suggesting a close relationship between METTL14 and the inflammatory response. The relationship between SAA and METTL14 has not yet been reported and the precise mechanisms underlying this association require further elucidation in future work. METTL14 was also observed to correlate with the Gensini score and higher numbers of coronary artery branches containing lesions, suggesting a relationship between METTL14 and CHD stenosis and severity. The above studies provide a stronger theoretical basis for the relationship between METTL14 and inflammation. Although studies have identified the role of METTL14 in CHD and its relationship with inflammatory markers, the biological effects of METTL14 on the initiation and progression of CHD have not been investigated. Combined with previous and our results, it is speculated that it is possible to delay the progression of CHD by intervening or inhibiting potential molecular targets. Nevertheless, there are still several shortcomings in this study. First, it was a single-center study with a small sample size, with some variation in the clinical baseline data of the study population, so a subsequent large-sample study is needed to further confirm these results. In addition, there are many risk factors for CHD and only a single inclusion criterion was used. Thus, although we observed a significant link between METTL14 levels and CHD risk, the use of METTL14 as a CHD biomarker requires further verification with large-sample and multi-center studies. Specific targeting is challenging in disease treatment and it is possible that the combination of transcription factors with targets may be useful, providing a stronger theoretical basis for the prevention of CHD.

## Data Availability

The raw data supporting the conclusions of this article will be made available by the authors, without undue reservation.
